# How to make a haploid male

**DOI:** 10.1002/evl3.107

**Published:** 2019-03-07

**Authors:** Laura Ross, Nicholas G. Davies, Andy Gardner

**Affiliations:** ^1^ Institute of Evolutionary Biology University of Edinburgh Edinburgh EH9 3JT United Kingdom; ^2^ Department of Infectious Disease Epidemiology London School of Hygiene and Tropical Medicine London WC1E 7HT United Kingdom; ^3^ School of Biology University of St Andrews Dyers Brae St Andrews KY16 9TH United Kingdom

**Keywords:** Kin selection, haplodiploidy, inbreeding, male heterogamety, population genetics, sex chromosome, sex determination

## Abstract

Haplodiploidy has evolved repeatedly among invertebrates, and appears to be associated with inbreeding. Evolutionary biologists have long debated the possible benefits for females in diplodiploid species to produce haploid sons–beginning their population's transition to haplodiploidy–and whether inbreeding promotes or inhibits this transition. However, little attention has been given to what makes a haploid individual male rather than female, and whether the mechanism of sex determination may modulate the costs and benefits of male haploidy. We remedy this by performing a theoretical analysis of the origin and invasion of male haploidy across the full range of sex‐determination mechanisms and sib‐mating rates. We find that male haploidy is facilitated by three different mechanisms of sex determination–all involving male heterogamety–and impeded by the others. We also find that inbreeding does not pose an obvious evolutionary barrier, on account of a previously neglected sex‐ratio effect whereby the production of haploid sons leads to an abundance of granddaughters that is advantageous in the context of inbreeding. We find empirical support for these predictions in a survey of sex determination and inbreeding across haplodiploids and their sister taxa.

**Impact Summary**

This article deals with an important outstanding question: why is reproduction, an essential feature of all living systems, so variable across the tree of life? Haplodiploidy––an unusual reproductive system in which females develop from fertilised (diploid) eggs in the usual way but males develop from unfertilised (haploid) eggs–is one of the most common alternative reproductive modes among animals, and has attracted huge and sustained attention from evolutionary biologists for decades, particularly in relation to its association with eusocial insect societies.

Evolutionary biologists have long debated the possible benefits for females in diplodiploid species to start producing haploid sons, and thereby begin their population's transition to haplodiploidy. However, little attention has been given to what makes haploid individuals male rather than female in the first place, and how the mechanism of sex determination affects the costs and benefits of male haploidy. Moreover, there has been a longstanding debate as to whether inbreeding tends to promote or inhibit the evolution of haplodiploidy, with the majority view tending to favour an inhibitory role for inbreeding, on theoretical grounds, despite an apparently strong positive association between inbreeding and haplodiploidy in nature.

We address these problems by means of a theoretical analysis of the evolution of male haploidy across the full range of sex‐determination mechanisms and inbreeding rates. This reveals that male haploidy is facilitated by three different mechanisms of sex determination and is impeded by others, and that inbreeding does not pose the evolutionary barrier that had previously been imagined. We also conduct an empirical survey of all known independent origins of haploidiploidy, finding support for our theoretical predictions.

Sexual reproduction, whereby each offspring receives genetic material from two parents, is by far the most common mode of reproduction across the animal kingdom. Yet, in a sizeable minority of extant animal species (∼12%), whilst daughters are produced sexually and receive a genome from both their mother and their father, sons are produced asexually from unfertilised eggs and have just one haploid genome that they derive from their mother (Normark 2003; la Filia et al. 2015; Blackmon et al. 2017). Such haplodiploid inheritance has evolved repeatedly and in a variety of different invertebrate clades, where it is often associated with gregarious broods and chronic inbreeding (Hamilton 1967, 1993; Normark 2004, 2006; Fig. 1A and B, Table S1).

**Figure 1:**
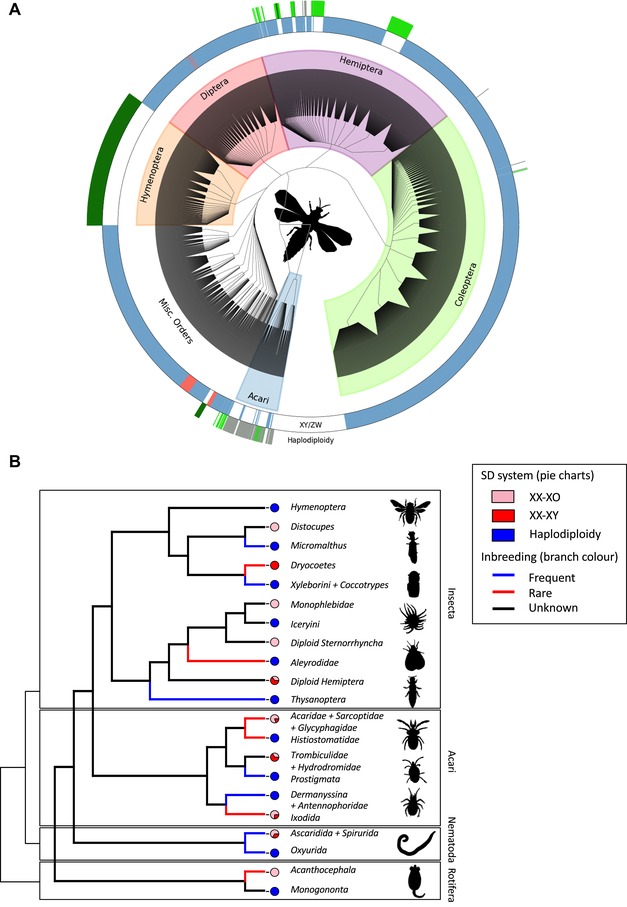
The phylogenetic distribution of haplodiploidy among invertebrates. (A) The distribution of genetic sex determination systems (GSDs) and haplodiploidy across invertebrates based on the Tree of Sex database (Tree of Sex Consortium 2014). The tree figure was adapted from Tree of Sex Consortium (2014), but only including those tips for which relevant SD data was available. The innermost ring depicts GSD systems (with male heterogamety in blue and female heterogamety in red). The outer ring depicts haplodiploid and related SD systems (with dark green depicting “true” haplodiploidy (arrhenotoky), light green depicting “Paternal genome elimination, PGE” where males develop from fertilized eggs but lose their paternal genome during development (Gardner and Ross 2014) and olive green depicting species with haploid males that could either result from PGE or arrhenotoky. (B) Cladogram of all haplodiploid (arrhenotokous) clades and their diploid sistergroups. Tree topology based on Misof et al. (2014), Blackmon et al. (2015), Johnson et al. (2018) and the Tree of Life (http://tolweb.org/tree/). Pie charts show the frequencies of different SD systems for each clade (see Table S1), with X0 in pink, XY in red and haplodiploidy in blue. Branch colour indicates if inbreeding is frequent (obligate, in blue), inbreeding is rare (facultative, in red) or inbreeding rate is unknown (black). More details and references in Table S1.

The transition from standard diplodiploid sexual reproduction to haplodiploidy requires that some mothers start producing haploid sons, and a long‐running strand of evolutionary theory has explored the possible benefits of doing so (Table 1). As the de novo appearance of the haploid condition is likely associated with substantial viability costs–which might be lessened under inbreeding on account of the purging of recessive deleterious alleles (Hartl and Brown 1970; Borgia 1980; Goldstein 1994; Smith 2000)–it appears there must also be substantial benefits if the transition to haplodiploidy is to be driven by natural selection. According to the “maternal transmission advantage” hypothesis, a female gains a twofold benefit from producing a haploid son instead of a diploid son, as all the genes he successfully transmits to future generations are derived from her, rather than half from her and half from her mate (Brown 1964; Hartl and Brown 1970; Bull 1979; Smith 2000). Whilst this advantage could potentially explain the evolution of haplodiploidy in outbred populations, the twofold benefit has been predicted to disappear as populations become increasingly inbred, because as a female becomes increasingly related to her mates, her preventing them from genetically contributing to her sons becomes increasingly pointless (Brown 1964; Bull 1979; Smith 2000). Alternatively, some theorists have suggested that haplodiploidy enables a level of control over sex allocation that is not enjoyed under diplodiploidy, and which might be particularly useful in the context of ecological factors–such as inbreeding–that favour biased sex ratios (Hamilton 1967; Borgia 1980; Bull 1983; Haig 1993; Normark 2004; Burt and Trivers 2006).

**Table 1:** An overview of adaptive hypotheses for the evolution of male haploidy

Benefits of haplodiploidy	Predictions under inbreeding	References	Notes
Maternal transmission advantage	Inbreeding inhibits haplodiploidy, because drive is only worthwhile in heterozygotes.	(Brown 1964; Bull 1979; Smith 2000)	This effect is captured in the present model.
Local mate competition I	Inbreeding promotes haplodiploidy, as it favours female bias, and haplodiploidy might enable maternal control of sex allocation.	(Hamilton 1967; Borgia 1980; Normark 2004)	Although mothers may control offspring sex in haplodiploid populations, this does not mean that a mother who chooses to produce a haploid offspring in an otherwise diploid population has much control over her offspring's sex ratio, as her diploid offspring are probably an equal mix of sons and daughters. Direct control of sex allocation is neglected in the present model.
Local mate competition II	Inbreeding promotes haplodiploidy, as inbreeding favours female bias, and producing haploid males might lead to female bias in subsequent generation.	(Bull 1983; Haig 1993; Burt and Trivers 2006)	Bull (apparently incorrectly) attributed this to Hamilton and Borgia, and dismissed it as lacking generality. This effect is captured in the present model.
Reduced mutation load	Unclear.	(Goldstein 1994)	Inbreeding was not considered in Goldstein's analysis. Deleterious mutations are neglected in the present model.
Maternally transmitted endosymbiont	Inbreeding promotes haplodiploidy, because inbreeding favours female bias, and haploid males–induced by endosymbiont in order to enhance its own transmission –might lead to a female bias in subsequent generation.	(Normark 2004; Kuijper and Pen 2010)	Endosymbionts are neglected in the present model.

However, whilst existing theory has considered the costs and benefits of producing a haploid male, relatively little attention has been given to the circumstances under which haploid individuals are expected to develop as males as opposed to females in the first place. That is, the sex determination (SD) mechanism employed by a given diplodiploid species may dictate whether it is even possible for that species to transition to haplodiploidy, irrespective of the costs and benefits involved. Moreover, whilst existing theory frames the mother's decision in terms of producing haploid sons versus diploid sons, of more relevance might be the tradeoff between producing haploid offspring versus diploid offspring, with the mother having little direct control of their sex. For example, if diploid offspring are equally likely to develop as sons or daughters, the production of haploid males might come at the expense of biasing the overall sex ratio toward males, with potential consequences for sex‐ratio selection. Furthermore, the SD mechanism will likely influence the sex of a haploid male's own offspring. For example, under classic haplodiploid inheritance, haploid males father only daughters and not sons. This too may have consequences for sex‐ratio selection, particularly in the context of an inbreeding lifestyle that favours female‐biased sex allocation.

Here, we investigate the origin and evolutionary invasibility of haploid males in diploid populations for a range of different SD mechanisms and sib‐mating rates. First, we identify which SD mechanisms–including different types of male (X0 and XY) and female (ZW and Z0) heterogamety–lead haploid individuals to develop as males versus females, with reference to published data on the molecular mechanisms underpinning SD in different taxonomic groups. Second, for those SD mechanisms that do robustly allow for the production of haploid males, we develop mathematical population models to quantify the likelihood of invasion of male haploidy from rarity, as a function of sib‐mating rate. Third, we compare the resulting theoretical predictions with the scant empirical data that exist in relation to mechanisms of SD across haplodiploids and their sister taxa, and consider the implications of our theoretical analysis for the longstanding puzzle as to whether inbreeding preceded or followed the evolution of haplodiploidy.

## Methods

### THEORETICAL ANALYSIS

First, we investigate how the SD mechanism employed in a diploid population will determine the sex of rare haploid individuals developing from unfertilised eggs. We exclude from our analysis those mechanisms of SD that are “environmental”, that is, where the haploid individual's genomic constitution does not determine its sex, but consider the full range of known–and unknown but feasible–“genetic” mechanisms of SD. Second, narrowing our attention to those SD mechanisms under which haploid individuals robustly develop as males, we investigate the invasibility of male haploidy in an evolutionary, population model.

We base our population model upon Gardner and Ross's (2014) analysis of whole genome elimination. Specifically, we assume an infinite number of patches, with each patch containing mated, adult females (foundresses) who lay eggs within that patch. Rather than specify the number of foundresses on each patch and their fecundities directly, we instead denote by *a* the probability that any two eggs chosen at random from the same patch were laid by the same foundress. In a departure from Gardner and Ross's (2014) model, we consider that although most eggs are fertilized and develop as viable diploid offspring with sex dependent on the details of the SD mechanism, a vanishing proportion of eggs remain unfertilized and develop as viable haploid male offspring with probability 1‐*c*, where *c* is the viability cost of haploidy. Viable offspring mate at random within their patch, with each female mating with a large number of males. Males then die, and females disperse to found new patches, returning the population to the beginning of the lifecycle.

The larger the viability cost *c* of haploidy, the more stringent will be the condition for natural selection to favour an increase in allocation to haploid males. Accordingly, the goal of our analysis is to determine the threshold haploid viability cost c*, such that natural selection favours an increase in allocation to haploid males when *c* < c* and favours a decrease in allocation to haploid males when *c* > c*. We may term this threshold viability cost c* the “potential for male haploidy” (cf Gardner 2010). By expressing the threshold viability cost c* in terms of SD mechanism and rate of inbreeding, we may determine how these factors modulate the benefit of male haploidy (see Box 1 and Supplementary Material for more details).

**Box 1. Inclusive fitness derivation**

We derive the potential for male haploidy c* when sex is determined by X‐chromosome count or a paternal‐origin X‐linked feminizer as follows (see Methods for model assumptions). Consider a population in which foundresses who produce haploid sons are vanishingly rare, and fasten attention upon a focal foundress who leaves a proportion δ of her eggs unfertilized. If the total number of eggs produced by all foundresses on her patch is *N* and her eggs constitute a proportion *x* of this total, then she gives rise to *m*_1_ = *Nx*δ(1‐*c*) viable haploid sons, *m*_2_ = *Nx*(1‐δ)/2 diploid daughters and *m*_3_ = *Nx*(1‐δ)/2 diploid sons, whilst her cofoundresses collectively produce *m*_4_ = *N*(1‐*x*)/2 diploid daughters and *m*_5_ = *N*(1‐*x*)/2 diploid sons. All of the female offspring produced on this patch experience exactly the same mating environment, with a proportion α = *m*_1_/(*m*_1_+*m*_3_+*m*_5_) of their mates being haploid sons of the focal foundress, a proportion β = *m*_3_/(*m*_1_+*m*_3_+*m*_5_) of their mates being diploid sons of the focal foundress, and a proportion γ = *m*_5_/(*m*_1_+*m*_3_+*m*_5_) of their mates being diploid sons of the cofoundresses.

Now consider a patch founded by a daughter of the focal foundress, and denote the number of eggs that she produces *Ny* and the number of eggs that her cofoundresses produce *N*(1‐*y*). This daughter of the focal foundress produces *n*_1_ = *Ny*α diploid daughters fathered by her haploid brothers, *n*_2_ = *Ny*β/2 diploid daughters fathered by her diploid brothers, *n*_3_ = *Ny*β/2 diploid sons fathered by her diploid brothers, *n*_4_ = *Ny*γ/2 diploid daughters fathered by unrelated diploid males and *n*_5_ = *Ny*γ/2 diploid sons fathered by unrelated diploid males, whilst the other foundresses on her patch collectively produce *n*_6_ = *N*(1‐*y*)/2 diploid daughters and *n*_7_ = *N*(1‐*y*)/2 diploid sons. The ratio of females to males on the patch is therefore *F* = (*n*_1_+*n*_2_+*n*_4_+*n*_6_)/(*n*_3_+*n*_5_+*n*_7_).

Finally, consider a patch founded by a daughter of a cofoundress of the focal foundress, and denote the number of eggs that she produces *Nz* and the number of eggs that her cofoundresses produce *N*(1‐*z*). This daughter of a cofoundress of the focal foundress produces *n*_8_ = *Nz*α diploid daughters fathered by haploid sons of the focal foundress, *n*_9_ = *Nz*β/2 diploid daughters fathered by diploid sons of the focal foundress, *n*_10_ = *Nz*β/2 diploid sons fathered by diploid sons of the focal foundress, *n*_11_ = *Nz*γ/2 diploid daughters fathered by diploid sons of a cofoundress of the focal foundress and *n*_12_ = *Nz*γ/2 diploid sons fathered by diploid sons of a cofoundress of the focal foundress, whilst the other foundresses on her patch collectively produce *n*_13_ = *N*(1‐*z*)/2 diploid daughters and *n*_14_ = *N*(1‐*z*)/2 diploid sons. The ratio of females to males on the patch is therefore *G* = (*n*_8_+*n*_9_+*n*_11_+*n*_13_)/(*n*_10_+*n*_12_+*n*_14_).

The inclusive fitness (Hamilton 1964) of the focal foundress may then be defined as *H* = E(*m*_2_(*n*_1_ *p*_1_ + *n*_2_ *p*_2_ + *n*_3_ *F p*_3_ + *n*_4_ *p*_4_ + *n*_5_ *F p*_5_ + *n*_6_ *p*_6_ + *n*_7_ *F p*_7_) + *m*_4_(*n*_8_ *p*_8_ + *n*_9_ *p*_9_ + *n*_10_ *G p*_10_ + *n*_11_ *p*_11_ + *n*_12_ *G p*_12_ + *n*_13_ *p*_13_)), where *p* denotes the consanguinity of the focal foundress to each type of grandoffspring and E denotes an expectation taken over the values *x*, *y*, and *z*. The consanguinities are given by *p*_1_ = (3+5*f*)/8, *p*_2_ = (1+3*f*)/4, *p*_3_ = (1+3*f*)/4, *p*_4_ = (1+3*f*)/8, *p*_5_ = (3+5*f*)/8, *p*_6_ = 0, *p*_7_ = 0, *p*_8_ = (1+*f*)/4, *p*_9_ = (1+3*f*)/8, *p*_10_ = (1+3*f*)/8, *p*_11_ = 0, *p*_12_ = 0, and *p*_13_ = 0, where *f* is the consanguinity between mating partners. Note that *f* = *a*((1/4) × (1+*f*)/2 + (1/2) × *f* + (1/4) × *f*), which rearranges as *f* = *a*/(8‐7*a*). Also note that E(*x*) = E(*y*) = E(*z*) = ∑*_i_*_= 1_ *^T^*(1/*T*)*x_i_* = σ and E(*x* ^2^) = E(*y* ^2^) = E(*z* ^2^) = ∑*_i_*_=1_ *^T^*(1/*T*)*x_i_* ^2^ = τ, where *x_i_* denotes the proportion of a patch's total egg production that derives from the *i* ^th^ foundress among the *T* foundresses that contribute eggs to the patch, and that τ/σ = *a*.

The focal foundress increases her inclusive fitness by increasing her investment into haploid sons if d*H*/dδ > 0, decreases her inclusive fitness by increasing her investment into haploid sons if d*H*/dδ < 0, and breaks even if d*H*/dδ = 0. Accordingly, the threshold viability cost c* that defines the potential for male haploidy satisfies d*H*/dδ|*_c_*_=_ _c__*_ = 0, and is given by c* = (2‐4*a*+5*a* ^2^‐2*a* ^3^)/(4‐5*a*+5*a* ^2^‐2*a* ^3^), as reported in the Results.

### EMPIRICAL SURVEY

To empirically assess our key theoretical predictions, we survey sex chromosome karyotype data available on the “Tree of Sex” database (Tree of Sex Consortium 2014) for the diploid sister groups of known haplodiploid clades. Haplodiploidy has evolved once within the Rotifera, once within the Nematoda, 8–12 times within the Acari and seven times within the Insecta. We determine the most likely diploid sister group based on published phylogenies for each clade (see Lohse and Ross 2015 and references in Table S1). For each of these sister groups we have collected available data on chromosome karyotype, which allows us to distinguish between male and female heterogametic systems. Sex chromosome karyotype cannot decisively distinguish between any of the five possible mechanisms underlying genomic SD we have considered above. In species where the Y or W chromosome is absent (X0 and Z0 systems), SD involving a dominant SD locus is unlikely, but the other four mechanisms are equally plausible. In contrast, in XY and ZW systems all five mechanisms could underlie this karyotype. However, it is generally assumed that the loss of a Y/W chromosome is less likely in dominant SD‐locus systems (Bachtrog et al. 2014; Blackmon and Demuth 2014), so frequent sex chromosome loss within a clade may indicate the presence of a counting, dosage, parent‐of‐origin, or CSD mechanism. We present the results of our survey in Fig. 1B and Table S1.

We also assess the likelihood that haplodiploidy evolved in the context of inbreeding. We have done so by combining information about the mating systems of haplodiploid clades as well as their diploid sistergroups. We surveyed a number of different aspects of species’ mating system (see Table S1 and Gardner and Ross 2014) that together allow us to classify each clade as being “frequently inbreeding”, “facultatively inbreeding” or “primarily outbreeding”. We find that inbreeding (either frequent or facultative) is common in most haplodiploid clades. It is currently unclear whether inbreeding was already present before the evolutionary transition towards haplodiploidy: our inference of inbreeding in the diploid sistergroups suggests most of these are either outbreeders or of unknown mating system. This suggests that mating systems involving frequent inbreeding may have evolved after the transition to haplodiploidy, but the long evolutionary history of haplodiploidy in most clades makes it challenging to draw any firm conclusions.

## Results and Discussion

### ONLY SOME SD MECHANISMS ALLOW THE RELIABLE PRODUCTION OF HAPLOID MALES

**Figure 2:**
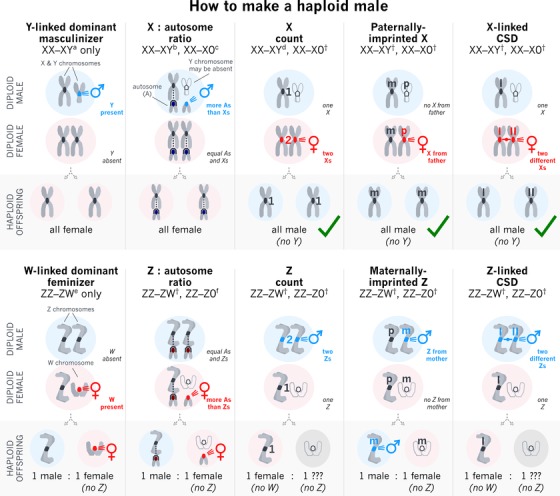
How to make a haploid male. The sex and viability of haploid offspring developing from unfertilized eggs under five alternative sex determination systems. Under male heterogamety (*top row*), unfertilized eggs reliably develop as males under the X count, Paternal‐origin X feminizer, and X‐linked CSD mechanisms (marked with green ticks). Under female heterogamety (*bottom row*), none of the five mechanisms reliably cause unfertilized eggs to develop as males. Haploid offspring may suffer from additional viability costs beyond the cost of haploidy per se, as follows: no *Y*, males lacking a Y chromosome under XX–XY (but not XX–X0) may incur a viability cost since males normally carry a Y chromosome in these species; *no W*, females lacking a *W* chromosome under ZZ–ZW (but not ZZ–Z0) may incur a viability cost since females normally carry a W chromosome in these species; *no Z*, individuals lacking a Z chromosome may incur a viability cost since individuals of either sex normally carry a Z chromosome in these species. Known examples of SD mechanisms are as follows: ^a^mammals, Diptera (*Aedes aegypti, Anopheles gambiae, Ceratitis capitata, Lucilia cuprina, Musca domestica*), Coleoptera (*Tribolium castaneum*), fish (*Oryzias latipes, Oryzias luzanensis, Odontesthes hatcheri, Oncorhynchus mykiss*) (Stuart and Mocelin 1995; Bachtrog et al. 2014; Hall et al. 2015; Krzywinska et al. 2016); ^b^ *Drosophila melanogaster* (disputed; Erickson and Quintero 2007), tiger pufferfish (*Takifugo rubripes*) (Bachtrog et al. 2014); ^c^ *Caenorhabditis elegans* (with XX individuals hermaphrodite, not female); ^d^ *D. melanogaster* (suggested; Erickson and Quintero 2007); ^e^silkworm (*Bombyx mori*) (Kiuchi et al. 2014); ^f^chicken (*Gallus gallus*), smooth tongue sole (*Cynoglossus semilaevis*) (Bachtrog et al. 2014); ^†^hypothetical/examples unknown. See main text for details of each mechanism.

Under what conditions are unfertilised, haploid eggs expected to develop as males rather than females (Fig. 2)? In the overwhelming majority of terrestrial arthropods, including all those that have given rise to haplodiploid clades (Fig. 1), an individual's sex is determined by the genomic material it receives from its parent(s), and so we restrict our attention to such “genetic” sex‐determination (GSD) mechanisms (Bull 1983; Bachtrog et al. 2014; Beukeboom and Perrin 2014). Here, we consider five different classes of possible SD mechanism.

First, sex might be determined by the presence versus absence of a sex‐linked dominant allele (Fig. 2). Under male heterogamety (XY) this must involve a Y‐linked masculinizer gene, which will be absent from haploid individuals developing from unfertilized eggs, making them female (Hartl and Brown 1970), not male. Under female heterogamety (ZW), a W‐linked feminizer gene will be involved. Accordingly, half of the haploid individuals will inherit a Z chromosome—making them male—but the other half will inherit a W chromosome—making them female, and likely inviable, owing to their lacking crucial Z‐linked genes that would normally be carried by both sexes (Bull 1983; Bachtrog et al. 2014).

Second, sex might be determined by sex‐chromosome/autosome balance (Fig. 2). Under male heterogamety (X0 and XY), diploid individuals carrying one set of X‐linked genes and two sets of autosomal genes have a “low” X:A ratio and develop as males, whilst individuals carrying two sets of X‐linked genes and two sets of autosomal genes have an “even” X:A ratio and develop as females. Accordingly, haploid individuals will carry one set of X‐linked genes and one set of autosomal genes (“even” X:A ratio) and hence will be female (Bridges 1930; Whiting 1945; Crozier 1971; Bull 1983; Miller et al. 1988; Erickson and Quintero 2007). Under female heterogamety (Z0 and ZW) diploid individuals carrying one set of Z‐linked genes and two sets of autosomal genes have a “low” Z:A ratio and develop as females, whilst individuals carrying two sets of Z‐linked genes and two sets of autosomal genes have an “even” Z:A ratio and develop as males. Accordingly, half of all haploid individuals will carry one set of Z‐linked genes and one set of autosomal genes (“even” Z:A ratio) and hence will be male, but the other half will carry no Z‐linked genes and one set of autosomal genes (“zero” Z:A ratio) and hence will probably be female (Whiting 1945; Bull 1983)—and likely inviable owing to their lacking crucial Z‐linked genes.

Third, X‐chromosome or Z‐chromosome count per se might determine sex (Fig. 2), which has been suggested for *Drosophila* (Erickson and Quintero 2007). This could perhaps involve either a threshold dose of an X or Z‐linked transcript being assessed at a set point in development, or the dose of X or Z‐linked transcripts being assessed relative to maternally derived transcripts (Erickson and Quintero 2007; Salz and Erickson 2010). Under male heterogamety (X0 and XY), diploid individuals carrying one X chromosome per cell develop as males, whilst individuals carrying two X chromosomes develop as females. Thus, haploid individuals will each carry one X chromosome per cell and hence will be male—although possibly inviable or infertile under XY owing to lack of Y‐linked genes crucial for male function. Under female heterogamety (Z0 and ZW), diploid individuals carrying one Z chromosome per cell would develop as females, whilst individuals carrying two Z chromosomes would develop as males. Thus, half of the haploid individuals will carry one Z chromosome per cell and hence would be female (and possibly inviable under ZW owing to lack of W‐linked genes crucial for female function), and the other half will carry no Z chromosomes, making their sex difficult to predict (and likely they would be inviable owing to lack of crucial Z‐linked genes).

Fourth, parent‐of‐origin effects could in principle determine sex (Fig. 2). Whilst such a mechanism has not been confirmed in any species, it has been suggested to underpin haplodiploid SD in a parasitoid wasp (Verhulst et al. 2010). Under male heterogamety (X0 and XY) diploid individuals carrying a paternal‐origin X^p^ chromosome have karyotype X^m^X^p^ and are female, whereas diploid individuals lacking a paternal‐origin X^p^ chromosome have karyotype X^m^0 or X^m^Y^p^ and are male. Haploid individuals will have karyotype X^m^ and hence will be male—although possibly inviable under XY owing to lack of Y‐linked genes crucial for male function. In contrast, under female heterogamety (Z0 and ZW) diploid individuals carrying a maternal‐origin Z^m^ chromosome have karyotype Z^p^Z^m^ and would be male whereas diploid individuals lacking a maternal‐origin Z^m^ chromsome have karyotype Z^p^0 or Z^p^W^m^ and would be female. Half of the haploid individuals will have karyotype Z^m^ and hence would be male, but the other half will have karyotype 0 or W^m^ and hence would be female (and likely inviable owing to lack of crucial Z‐linked genes, and possibly also under ZW owing to lack of W‐linked genes crucial for female function).

Finally, another possible scenario involves X/Z‐linked complementary SD (X‐CSD/Z‐CSD; Fig. 2). In this scenario, there are many alleles segregating at an X‐ or Z‐linked SD locus, and the sex of an individual is determined by whether they carry two different SD alleles or just one—in the latter case either (i) because they are hetero‐ or hemigametic, and in possession of only one X or Z chromosome, which means they can only carry one allele at the SD locus, or (ii) because they have two X or Z chromosomes but are homozygous at the SD locus. Such a SD mechanism is currently unknown among diplodiploids but has strong similarities to autosomal‐SD mechanisms found in Hymenoptera (Bull 1983; Heimpel and de Boer 2008). For example, in the honey bee (*Apis mellifera)*, “femaleness” is determined by heterozygosity at the *csd* locus directing female‐specific splicing of the *fem* gene (Beye et al. 2003; Hasselmann et al. 2008). Furthermore, autosomal CSD is so widespread among hymenopteran insects that it could conceivably pre‐date the evolution of haplodiploidy (Heimpel and de Boer 2008) and have been present in their diploid ancestors. In the context of male heterogamety (X0 and XY) we assume that diploid individuals with genotype xx (i.e. the same allele x present in two copies) or x (i.e. a single allele x present in one copy) at an X‐linked CSD locus are male, whereas diploid individuals with genotype xx′ (i.e. two alleles x and x′ each present in one copy) are female. Haploid individuals will have genotype x and hence will be male—although possibly inviable under XY owing to lack of Y‐linked genes crucial for male function. In contrast, in the context of female heterogamety (Z0 and ZW), diploid individuals with genotype zz or z at a Z‐linked CSD locus would be female whereas diploid individuals with genotype zz′ would be male. Accordingly, half of the haploid individuals will have genotype z and hence would be female (and possibly inviable under ZW owing to lack of W‐linked genes crucial for female function), and the other half will have a null genotype and hence their sex is difficult to predict (and they would likely be inviable owing to lack of crucial Z‐linked genes).

In summary, only three of the above mechanisms of SD–X‐chromosome counting, paternal‐origin X‐linked feminizer and X‐linked CSD–appear to have unfertilized eggs robustly developing as haploid males, and all three involve male heterogamety.

### INBREEDING DOES NOT PRESENT AN OBVIOUS BARRIER TO MALE HAPLOIDY

**Figure 3:**
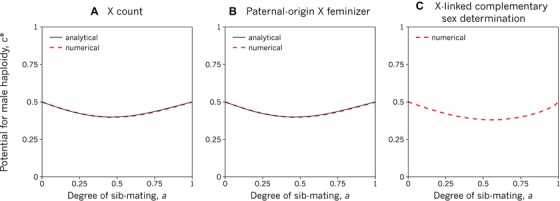
Inbreeding does not present an obvious barrier to the evolution of male haploidy. Natural selection favours the invasion of male haploidy when the viability cost of haploid males is less than c*, which varies with the degree of sib‐mating and the mechanism of sex determination (SD). For the three SD systems under which unfertilised eggs reliably develop into males (see Fig. 2), inbreeding does not strongly inhibit male haploidy. The SD systems shown here are (A) X count, where individuals are female if they have two X chromosomes and are otherwise male; (B) paternal‐origin X feminizer, where individuals are female if they inherit an X chromosome from their father and are otherwise male; and (C) X‐linked complementary sex determination (CSD), where individuals are female if they are heterozygous at an X‐linked CSD locus and male if they are homozygous or hemizygous at this locus. Numerical and, where possible, analytical results are shown (see Methods). Note that panels (A) and (B) are identical.

We now ask under which circumstances natural selection is expected to favour an increase in allocation to haploid males, and how this is modulated by inbreeding, for each of the three SD mechanisms identified above under which haploid males robustly develop from unfertilized eggs. Our mathematical model (see Methods) assumes a patch‐structured diplodiploid population, in which the rate of sib‐mating is described by a parameter *a*, and we use this to calculate the fitness consequences for a mother who produces a small number of haploid offspring among her brood. The key output of the model is a real‐valued “potential for male haploidy” c*, representing the maximum viability cost associated with the haploid condition that nevertheless permits male haploidy to be favoured by natural selection. The scenarios in which SD is by X‐chromosome counting or a paternal‐origin X‐linked feminizer are formally equivalent in terms of their evolutionary dynamics, and yield the following potential for male haploidy:

$$c^{*}=\frac{2-4a+5a^{2}-2a^{3}}{4-5a+5a^{2}-2a^{3}}$$

(see Box 1 for derivation). This equation describes a U‐shaped relationship between rate of inbreeding and potential for male haploidy (Fig. 3A and B). Specifically: in the absence of sib‐mating (*a* = 0), the potential for male haploidy is c* = ½, recovering Bull's (1979) prediction that natural selection favours male haploidy so long as the viability cost does not exceed one half; an intermediate level of sib‐mating (0 < *a* < 1) leads to a slightly reduced potential for male haploidy (c* < ½; with a minimum of c* ≈ 0.40 at *a* ≈ 0.46); and under full sib‐mating (*a* = 1), the potential for male haploidy is c* = ½. The complexity of the X‐linked CSD scenario means that it resists analytical solution, but numerical analysis–performed under the assumption that males homozygous at the SD locus are inviable (see Supplementary Material)–reveals a very similar U‐shaped relationship: in the extremes of absent or full sib‐mating (*a* = 0 or 1), the potential for male haploidy is c* = ½, while an intermediate level of sib‐mating (0 < *a* < 1) leads to a reduced potential for male haploidy (c* < ½; with a minimum of c* ≈ 0.38 at *a* ≈ 0.56; Fig. 3C).

Why is the potential for male haploidy under full inbreeding exactly the same as in a fully outbred population? Under full sib‐mating (*a* = 1), a mother gains no transmission advantage from haploid male production (since she is genetically identical to her mates), nor does she lose grandoffspring owing to haploid‐male inviability among her sons (since her surviving sons inseminate her daughters just as surely). Instead, leaving some eggs unfertilized reduces her production of daughters (since females never develop from unfertilized eggs) while increasing the rate at which her remaining daughters produce daughters of their own (since her haploid sons only sire daughters), and these two effects exactly cancel when *c* = 1/2 (see Supplementary Material). That is, inbreeding does not necessarily inhibit the evolution of male haploidy.

### X0 MALES AND INBREEDING ARE ASSOCIATED WITH THE ORIGINS OF HAPLODIPLOIDY

How do these theoretical predictions compare with empirical observation? Comparative empirical data on the molecular mechanisms of SD across taxonomic groups relevant to the evolution of male haploidy are scant (Blackmon et al. 2017), such that they cannot be brought to bear upon the detailed predictions emerging from our theoretical analysis. The only molecular insights into haplodiploid sex determination come from a small number of species within the Hymenoptera (e.g., Hasselmann et al. 2008; Verhulst et al. 2010), while no data is available for any other haplodiploid clade. However, two broad predictions may be assessed in light of the empirical–mainly karyotype–data that are available. First, our theoretical analysis suggests that mechanisms of SD involving male heterogamety (X0 and XY) relatively promote, and those involving female heterogamety (Z0 and ZW) relatively inhibit, the evolution of male haploidy. Second, our analysis suggests that the X0 form of male heterogamety relatively promotes, whilst the XY form of male heterogamety relatively inhibits, the evolution of male haploidy, on account of male function in the latter system becoming evolutionarily reliant upon Y‐linked genes that cannot be passed from mother to haploid son, and dominant Y‐linked sex determiners resulting in haploid offspring developing as female rather than male. Surveying the sex chromosome karyotype data available on the “Tree of Sex” database (Tree of Sex Consortium 2014) for the diplodiploid sister groups of known haplodiploid clades (Fig. 1B; Table S1), we find support for both of these predictions: the diplodiploid ancestors of all haplodiploid clades (i) were male heterogametic and (ii) most likely did not employ a Y‐linked dominant SD gene, as the vast majority were either exclusively X0 (six out of the eight origins for which we have data) or a mixture of X0 and XY. The survey also makes clear that inbreeding is common in all haplodiploid clades, but cannot resolve whether inbreeding preceded or followed the evolution of male haploidy.

### HOW TO MAKE A HAPLOID MALE

Heritable variation is the fuel that drives the process of adaptation, and natural selection can only work with the raw variation that it has at its disposal. Accordingly, if genetic and developmental systems introduce biases in the heritable variation that is generated, then predictable patterns of evolution may be discernable even before the logic of natural selection is brought to bear upon a given problem. Here, we have shown that taxonomic patterns of male haploidy may be, in part, driven by ancestral mechanisms of SD employed in a diplodiploid context. Specifically, we have found that haploid individuals are not expected to robustly develop as males under any form of female heterogamety (i.e. Z0 and ZW systems), but that they may do so under some forms of male heterogamety (i.e. X0 and XY systems)–which might itself be viewed as a partial form of male haploidy, as males are haploid with respect to their X‐linked genes. Despite the scarcity of relevant data, we have found support for this theoretical prediction in our empirical survey.

Moreover, we have suggested that an X0 system of male heterogamety might be particularly conducive to the evolution of male haploidy, as in XY systems haploid males would lack any Y‐linked genes that are crucial for male function. For example, *Drosophila* males that lack a Y chromosome are viable but sterile (Brosseau 1960). Again, despite the scarcity of data, we have found support for this prediction in our empirical survey. Note that whilst Y‐chromosomal material may be required for male function in some taxa, it is not required for viability in general for any taxon, as females who lack it remain fully viable. The same applies to W‐chromosomal material in ZW systems of female heterogamety, which might conceivably be essential for female function but is not essential for viability in general as males who lack it remain viable. However, the same is not true for X and Z material which, in their corresponding taxa, are carried by all individuals (and tend to contain many more genes than the Y and W) and hence may be more necessary for viability. Accordingly, whilst females in male heterogametic taxa are guaranteed to transmit an X chromosome to each of their haploid offspring, females in female heterogametic taxa are expected to transmit Z chromosomes to only half of their haploid offspring, which is likely associated with a large viability cost of haploidy and represents a further barrier to the evolution of haploid individuals in these taxa (Whiting 1945).

Our analysis has highlighted that knowing the precise molecular mechanism underpinning male heterogamety is crucial for predicting the sex of a haploid individual developing from an unfertilised egg and, accordingly, a taxon's predisposition to male haploidy. Our empirical survey of SD mechanisms in the diplodiploid species most closely related to haplodiploids shows that haplodiploidy almost certainly evolved in the context of male heterogamety (most likely X0). Sadly, there appears to be no information about the actual mechanism of SD in any X0 species (Beukeboom and Perrin 2014). Furthermore the lack of knowledge of sex determining pathways in haplodiploid taxa themselves, especially outside of the Hymenoptera, hinders our understanding about what might have been the mechanism in the diploid ancestor. As a result we have necessarily had to be speculative about the space of possibilities. In fact the exact molecular mechanism of SD is only known for a handful of animal species (Bachtrog et al. 2014; Beukeboom and Perrin 2014)–none of which occur in taxonomic groups giving rise to haplodiploidy–so it seems likely that many such mechanisms remain to be uncovered. In diploid insects and nematodes, the known molecular mechanisms underlying GSD are either based on dominant masculinizing/feminizing genes (such as the Y‐linked Yob gene in *Anopheles gambiae* (Krzywinska et al. 2016) and the Z‐linked fem gene in *Bombyx mori* (Kiuchi et al. 2014)) or X:autosome ratio (found in the nematode genus *Caenorhabditis* (Miller et al. 1988) and originally suggested for the X‐linked XSE genes in *Drosophila* (Bridges 1930; Salz and Erickson 2010)). Recent work suggests that SD in *Drosophila* might be based on absolute rather than relative dose of XSE: when the XSE dose reaches a certain threshold by the 12th embryonic division this activates the *Sxl* gene and initiates female development. However, haploid embryos still develop as females, as haploidy slows down embryonic development enough to allow the threshold XSE dose to accumulate (Erickson and Quintero 2007). So, none of the mechanisms described in insects and nematodes have unfertilized eggs robustly giving rise to haploid males.

Having narrowed the space of possibilities to three SD mechanisms that lead haploid individuals to develop as males, we considered the subsequent evolutionary dynamics with a particular focus on how the invasion success of haploid males is modulated by sib‐mating. The haploid condition is initially expected to be associated with substantial viability costs–as recessive deleterious mutations are exposed in haploid males and male fertility is likely reduced during the transition from meiotic to mitotic spermatogenesis–and so there has been a longstanding search for compensating benefits. Previously, the maternal‐transmission‐advantage hypothesis has emphasised the benefit accruing to a female who produces a haploid as opposed to a diploid offspring owing to her monopolising her offspring's reproductive value, rather than sharing half of this with her mate, and this benefit diminishes when mating partners are genetically related (Brown 1964; Bull 1979; Smith 2000). However, a possibly countervailing evolutionary pressure may arise owing to sex‐ratio selection which, in the context of inbreeding, is often expected to favour a female‐biased sex allocation. Bull (1983) briefly considered–but thought of little importance–the possibility that such sex‐ratio selection could have a male‐haploidy promoting effect, as the offspring of haploid males are–at least in extant haplodiploid taxa–all female (in our model, this follows directly from ancestral male heterogamety, whereby all haploid‐male sperm are X‐carrying).

Our analysis reveals how these two opposing effects of inbreeding quantitatively balance out in the context of X‐chromosome counting, a paternal‐origin X‐linked feminizer or X‐linked CSD. We have found that, in contrast to the expectation of many researchers who have investigated this topic, inbreeding does not necessarily inhibit the evolution of male haploidy, and indeed male haploidy appears to evolve just as readily under full sib‐mating as it does in a fully outbred population. One aspect of inbreeding not explicitly captured in our model is the concomitant purging of recessive deleterious alleles, which could reduce the viability cost incurred by haploid males, further promoting the transition to haplodiploidy. That is, inbreeding could alter the stringency of the condition *c* < c* for male haploidy to be evolutionary favoured either by altering the threshold viability cost c* (as explored in the present analysis) or else by altering the viability cost *c* itself (as explored in the simulation study of Smith 2000). In line with this prediction, we find in our empirical survey unequivocal support for the idea that inbreeding is often associated with haplodiploidy. Our analysis cannot determine which came first, but it clearly repudiates the entrenched view that inbreeding must have come second because it inhibits the evolution of haplodiploidy.

Associate Editor: K. Lythgoe

## Supporting information

Supplementary Material

**Table S1**. Overview of all taxonomic groups with haplodiploidy and their diploid sister clades.

## Data Availability

All data supporting the results are reported in supplementary Table S1.
