# Supplementary material for: How to make a haploid male
Source: Evol Lett. 2019 Mar 7;3(2):173–84. doi: 10.1002/evl3.107 (PMC6591549; doi:10.1002/evl3.107)
Supplement: Supplementary file 1 — Supplementary Material [file EVL3-3-173-s001.docx]

Supplementary material for:

**How to make a haploid male**

**1. Why is the potential for male haploidy the same under full sib-mating and full outbreeding?**

As predicted by Bull (1979), the viability cost incurred by haploid males must be less than one half for male haploidy to be favoured by natural selection under full outbreeding, because a cost of one half exactly balances the twofold transmission advantage enjoyed by mothers who produce haploid sons from unfertilized eggs. We show in the main text that as the rate of sib-mating *a* increases from zero, the potential for male haploidy follows a U-shaped curve, such that under full sib-mating (*a* = 1) the threshold viability cost – which we term the “potential for male haploidy” (cf Gardner 2010) – is again *c** = 1/2.

To see why the potential for male haploidy is *c** = 1/2 under full sib-mating, first consider the case in which sex is determined by X-chromosome count or by a paternal-origin X-linked feminizer. In this scenario, when a mother leaves a fraction δ of her *N* eggs unfertilized (assume δ is small), she produces *N*δ/2 fewer daughters than she would otherwise (since unfertilized eggs never develop into daughters), but a fraction ≈2δ(1–*c*) of those daughters’ mates (i.e., their brothers) are now haploid, so each remaining daughter herself produces *N*δ(1–*c*) more daughters than she would otherwise (since haploid males can only sire daughters, not sons). This results in a net gain of granddaughters when *N*δ/2 < *N*δ(1–*c*), or *c* < 1/2, and hence the potential for male haploidy under full sib-mating is *c** = 1/2. (It is not necessary to consider the gain or loss of grandsons, because under full sib-mating, males are superfluous beyond the point at which there are enough to inseminate females on a given patch.)

A similar logic applies when sex is determined by X-linked CSD under full inbreeding. In this scenario, a mother who leaves a small proportion δ of her *N* eggs unfertilized produces *N*δ/4 fewer daughters (and *N*δ/4 fewer inviable XX homozygotes) than she would otherwise, but a fraction ≈2δ(1–*c*) of those viable daughters’ mates are now haploid, so each remaining daughter herself produces *N*δ(1–*c*)/2 more daughters than she would otherwise (since the haploid males sire viable daughters and inviable XX homozygotes with equal probability). This results in a net gain of granddaughters for the foundress when *N*δ/4 < *N*δ(1–*c*)/2, or *c* < 1/2.

**2. Numerical simulations**

**Sex determination by X-chromosome count or a paternal-origin X-linked feminizer.** We simulate a large patch-structured population with non-overlapping generations. Mating occurs entirely within patches; after mating, males die and mated females disperse to found new patches. To model the effects of inbreeding and local mate competition, we assume that a proportion *a* of patches are founded by a single mated female, while a proportion 1 – *a* of patches are founded by a large number of mated females, such that *a* is the proportion of patches where sib-mating occurs.

For each individual, we focus on two loci: an autosomal locus which determines, if the individual is female, the proportion of her eggs that she leaves unfertilized; and a sex-linked locus which determines the sex of the individual at birth. We notate the autosomal locus as aa, Aa, or AA for diploid individuals and as a or A for haploid individuals, where a is the wild-type allele and A is the mutant allele that induces parthenogenesis in females.

We keep track of all “mated foundress types” in the population, such that *f­_i_*_,_*_g_* is the frequency of mated foundress type *i* in generation *g*. For brevity, we omit the subscript *g* henceforth. A mated foundress type is defined by the genotype of a mated female and the genotypes of the sperm she is storing. Note that the number of mated foundress types may be very large and may grow from one generation to the next, and that we make no attempt to specify which mated foundress types may be present in a given generation except by actually running the simulation. In general, mated foundress type *i* is defined by a female with *b­_i_* copies of the A allele (where *b­_i_* is 0, 1, or 2) and the relative proportions of her stored haploid sperm, as follows: a fraction *s_i_*_|a/X_ of her stored sperm is of genotype a/X; a fraction *s_i_*_|a/Y_ of her stored sperm is of genotype a/Y; a fraction *s_i_*_|A/X_ of her stored sperm is of genotype A/X; and a fraction *s_i_*_|A/Y_ of her stored sperm is of genotype A/Y, where *s_i_*_|a/X_ + *s_i_*_|a/Y_ + *s_i_*_|A/X_ + *s_i_*_|A/Y_ = 1. We assume that a randomly-selected patch has a probability *af_i_* of being founded by a mated foundress of type *i* and a probability 1 – *a* of being founded by a large number of mated foundresses, who follow the same distribution as that found in the population more broadly (i.e., on each patch founded by multiple foundresses, the frequency of mated foundresses of type *i* is *f_i_*).

A mated foundress of type *i* leaves a proportion δ = 0.001 of her eggs unfertilised if *b_i_* = 1 and a proportion 2δ of her eggs unfertilised if *b_i_* = 2, otherwise fertilising all of her eggs (i.e. we assume that the A allele acts additively). Of the unfertilised eggs, a proportion (1 – *c*)(1 – *b_i_*/2) develop into viable haploid males of genotype a/X and a proportion (1 – *c*)*b_i_*/2 develop into viable haploid males of genotype A/X, while the remaining proportion are inviable. Of the fertilised eggs, a proportion (1 – *b_i_*/2)*s_i_*_|a/X_ develop into females of genotype aa/XX; a proportion (1 – *b_i_*/2)*s_i_*_|A/X_ + (*b_i_*/2)*s_i_*_|a/X_ develop into females of genotype Aa/XX; a proportion (*b_i_*/2)*s_i_*_|A/X_ develop into females of genotype AA/XX; a proportion (1 – *b_i_*/2)*s_i_*_|a/Y_ develop into males of genotype aa/XY; a proportion (1 – *b_i_*/2)*s_i_*_|A/Y_ + (*b_i_*/2)*s_i_*_|a/Y_ develop into males of genotype Aa/XY; and a proportion (*b_i_*/2)*s_i_*_|A/Y_ develop into males of genotype AA/XY. Mating occurs randomly within patches, with each female mating multiple times, such that each female stores an unbiased sample of sperm from all males on the patch. After mating, males die, and mated females disperse to found new patches, restarting the life cycle; these mated females constitute the “mated foundress types” for the next generation of the simulation, thus providing the *b_i_* and *s_i_*_|*_ values for a new round of reproduction and mating.

We begin by allowing the population to reach equilibrium in the absence of the A allele: that is, we begin with a single “mated foundress type” for which *f_i_* = 1, *b_i_* = 0, *s_i_*_|a/X_ = *s_i_*_|a/Y_ = 1/2, and *s_i_*_|A/X_ = *s_i_*_|A/Y_ = 0, and iterate the population for 1000 generations. We then introduce the A allele at low frequency, mutating every a allele to an A with probability ι = 1x10^-6^. For example, suppose that the number of mating types after 1000 generations is *n*, and mated foundress types are indexed by *i* = {1, 2, ..., *n*}. After the mutation step, we will have 3*n* mated foundress types and new values *f’*, *b’*, and *s’* according to the following pseudocode:

for all 1 ≤ *i* ≤ *n*: *// females*

*f’_i_* ← (1 - ι)^2^ *f_i_* *// aa*

*f’_n+i_* ← 2ι *f_i_* *// Aa*

*b’_n+i_* ← 1

*f’*_2_*_n+i_* ← ι^2^ *f_i_ // AA*

*b’*_2_*_n+i_* ← 2

for all 1 ≤ *i* ≤ 3*n*: *// sperm*

*s’_i_*_|a/X_ ← (1 - ι) *s_i_*_|a/X_

*s’_i_*_|A/X_ ← ι *s_i_*_|a/X_

*s’_i_*_|a/Y_ ← (1 - ι) *s_i_*_|a/Y_

*s’_i_*_|A/Y_ ← ι *s_i_*_|a/Y_

Once the A allele is introduced, the number of mating types increases rapidly each generation, such that it is only possible to carry the simulation forward for a limited number of generations before exhausting computer resources. We carried forward the simulation from the introduction of the A allele for 18 generations, considering the A allele to have invaded if its average frequency among females Σ*_i_* *f_i_ b_i_* at the end of the simulation was higher than its frequency at its introduction.

This invasion process is carried forward for particular values of *a* and *c*. For a given value of *a*, we first verify that the allele invades when *c* = 0 and does not invade when *c* = 1. Accordingly, we assume that a threshold value of *c** exists between *c* = 0 and *c* = 1. We begin by setting *c*_0_ = 0 and *c*_1_ = 1, then test a new invasion for *c*_1/2_ = (*c*_0_ + *c*_1_)/2. If the allele invades for this value of *c*, we assume that the threshold lies between *c*_1/2_ and *c*_1_, and set *c*_0_ ← *c*_1/2_; otherwise, we assume that it lies between *c*_0_ and *c*_1/2_, and set *c*_1_ ← *c*_1/2_. We bifurcate the space between *c*_0_ and *c*_1_ ten times, such that finally we have a range for *c** of size 1/1024. We take the midpoint of this range, (*c*_0_ + *c*_1_)/2, as our estimate for *c** for a given value of *a*.

**X-linked complementary sex determination.** In this scenario, we assume that an individual carrying one allele at the X-linked SD locus (either in a single copy, in the case of a hemizygous haploid or diploid offspring lacking a paternal-origin X; or in two copies, in the case of a homozygous diploid offspring that has inherited the same allele from both parents) will, if viable, develop as a male, and an individual carrying two alleles at this locus (that is, a heterozygous diploid offspring that has inherited different alleles from either parent) will develop as a female. We assume that there is a very large number of alleles, all segregating at very low frequency, at the SD locus. One potential problem with X-linked complementary SD is that sibmating affects offspring sex directly through increasing the frequency of homozygotes. As a result a significant proportion of XX individuals might develop as males rather than females. For simplicity, we assume that homozygote XX males are infertile, as is generally the case for homozygous males under haplodiploid CSD (van Wilgenburg *et al*. 2006; Heimpel & de Boer 2008), and that they do not contribute to mating.

The numerical simulations are similar to those described above for X-chromosome count or a paternal-origin X-linked feminizer, except: (1) we track six sperm genotypes, instead of four, for each mated female type: a/S, a/D, a/0, A/S, A/D, and A/0, where the S stands for a CSD allele which is the same as one of the female’s two CSD alleles, the D stands for a CSD allele which is different from both of the female’s two CSD alleles, and the 0 stands for a null CSD allele; and (2) we differentiate between D-daughters and S-daughters on a patch, where D-daughters have inherited a D allele from their father and S daughters have inherited an S allele from their father. Individuals with two functional CSD alleles are viable females if the CSD alleles are different, and inviable XX males if the CSD alleles are the same; individuals with one functional CSD allele (and, optionally, one null CSD allele) develop as viable diploid or haploid males. Specifically, while the production of haploid males on a given patch is the same as detailed above, a mated female of type *i* produces (1 – *b_i_*/2)*s_i_*_|a/S_/2 genotype-aa S-daughters; (1 – *b_i_*/2)*s_i_*_|A/S_/2 + (*b_i_*/2)*s_i_*_|a/S_/2 genotype-Aa S-daughters; (*b_i_*/2)*s_i_*_|A/S_/2 genotype-AA S-daughters; (1 – *b_i_*/2)*s_i_*_|a/D_ genotype-aa D-daughters; (1 – *b_i_*/2)*s_i_*_|A/D_ + (*b_i_*/2)*s_i_*_|a/D_ genotype-Aa D-daughters; (*b_i_*/2)*s_i_*_|A/D_ genotype-AA D-daughters; (1 – *b_i_*/2)*s_i_*_|a/0_ genotype-aa sons; (1 – *b_i_*/2)*s_i_*_|A/0_ + (*b_i_*/2)*s_i_*_|a/0_ genotype-Aa sons; and (*b_i_*/2)*s_i_*_|A/0_ genotype-AA sons. Note that all S-daughters carry both of their mother’s CSD alleles, since: (i) their paternally-inherited CSD allele is, by definition, the same as one of their mother’s two CSD alleles; and (ii) their maternally-inherited CSD allele has to be equal to their mother’s *other* CSD allele, since if it was the *same* CSD allele as the S-daughter’s paternally-inherited CSD allele, the S-daughter would be an inviable XX male, not a daughter.

**References**

Bull, J. (1979). An advantage for the evolution of male haploidy and systems with similar genetic transmission. *Heredity* 43:361–381.

Gardner, A. (2010). Sex-biased dispersal of adults mediates the evolution of altruism among juveniles. *Journal of Theoretical Biology* 262:339–345.

Heimpel, G.E. & de Boer, J.G. (2008). Sex determination in the hymenoptera. *Annu. Rev. Entomol.*, 53, 209–230.

van Wilgenburg, E., Driessen, G. & Beukeboom, L.W. (2006). Single locus complementary sex determination in Hymenoptera: an “unintelligent” design? *Frontiers in Zoology 2006 3:1*, 3, 1.
