# Supplementary material for: How to make a haploid male
Source: Evol Lett. 2019 Mar 7;3(2):173–84. doi: 10.1002/evl3.107 (PMC6591549; doi:10.1002/evl3.107)
Supplement: Supplementary file 2 — Table S1. Overview of all taxonomic groups with haplodiploidy and their diploid sister clades. [file EVL3-3-173-s002.docx]

**Table S1 | Overview of all taxonomic groups with haplodiploidy and their diploid sister clades.** SD system for both the haplodiploid clades and their sister group was inferred from karyotype data available in the tree of sex database http://treeofsex.org^1^. Inbreeding inferred from *F*_IS_ estimates, mating-system observations and data on offspring sex-ratios and classified as “frequent inbreeding”, “facultative inbreeding” or “predominantly outbreeding”. Inbreeding classification based on several lines of evidence are indicated in the table using the following abbreviations: **WM** wingless males; **WF** wingless females; **SB** female-biased sex ratios; **PG** population-genetic evidence of low genetic diversity and excess homozygosity; **PS** life history leading to strong meta-population structure (“colonizers”); **SM** frequent sib-mating; **PMD** post-mating dispersal. Notes: ^*^Haplodiploidy has evolved at least twice within the Mesostigmata^2^, but there is no data on sex determination of any of the diploid members of the order. We therefore use the next closes outgroup Ixodida for comparison. ^**^The sistergroup is so large and diverse that it is impossible to reconstruct the ancestral SD karyotype/inbreeding level. ^***^Y chromosome very small, no recombination between X and Y^3^. ^****^The closest sister clade reproduces through parthenogenesis, so data on SD systems was obtained from the next closest outgroup

| Higher taxonomic group | Haplodiploid clade | SD system | Inbreeding | Sister clade | SD system (♯ of species with data) | Inbreeding | References |
| --- | --- | --- | --- | --- | --- | --- | --- |
| Acari: Acariformes | Histiostomatidae | unknown | predominantly outbreeding (absence of PMD) | Acaridae + Sarcoptidae + Glycyphagidae +Pyroglyphidae | XY (3) / XO (12) | predominantly outbreeding (absence of PMD) | ^2,4^ |
| Acari: Acariformes | Haplodiploid Prostigmata | unknown | frequently inbreeding | Trombiculidae + Hydrodromidae | XY (2) / XO (1) |  | ^2,4^ |
| Acari: Parasitiformes | Dermanyssina + Antennophoridae* | unknown | frequently inbreeding (PMD, PS, SB, SM) | Ixodida | XO (56) / XY (20) | predominantly outbreeding (PG) | ^2,4^ |
| Insecta | Hymenoptera | CSD | mixed (clades with both frequently inbreeding and predominantly outbreeding) | Non-hymenopteran Holometabolous insects | mixed** | mixed2 | ^5-7^ |
| Insecta | Thysanoptera | unknown | facultatively inbreeding (PG, SB, SM) | Hemiptera | XO (155) / XY (284) | unknown | ^8-10^ |
| Insecta: Coleoptera | Micromalthus | unknown | frequently inbreeding (combined with cyclic parthenogenesis) | Distocupes | XO (1) | unknown |  |
| Insecta: Coleoptera | Xyleborini + Coccotrypes | unknown | frequently inbreeding (WM, SB, PS, SM, PMD) | Dryocoetes | XY (2)*** | predominantly outbreeding | ^11,12^ |
| Insecta: Hemiptera | Aleyrodidae | unknown | predominantly outbreeding (absence WM and WF) PG | Sternorrhyncha | XO (13) | mixed | ^13^ |
| Insecta: Hemiptera | Iceryini**** | unknown | unknown but frequently selfing in the hermaphroditic members of the clade | Monophlebidae | XO (5) | unknown | ^14-16 Ross et al. in prep.^ |
| Nematoda | Oxyurida | unknown | frequently inbreeding (PS, SM, SB | Ascaridida + Spirurida | XO (26) / XY (13) | predominantly outbreeding | ^17,18^ |
| Rotifera | Monogononta | unknown | unknown but haplodiploidy occurs in the context of cyclic parthenogenesis | Acanthocephala | XO (8) | predominantly outbreeding | ^19^ |

References

1. Tree of Sex Consortium. Tree of Sex: A database of sexual systems. *Scientific Data* (2014).

2. Blackmon, H., Hardy, N. B. & Ross, L. The evolutionary dynamics of haplodiploidy: Genome architecture and haploid viability. *Evolution* **69,** 2971–2978 (2015).

3. Blackmon, H. & Demuth, J. P. Estimating Tempo and Mode of Y Chromosome Turnover: Explaining Y Chromosome Loss With the Fragile Y Hypothesis. *Genetics* **197,** 561–572 (2014).

4. Norton, R. A., Kethley, J. B., Johnston, D. E. & O'Connor, B. M. in (eds. Wrensch, D. L. & Ebbert, M. A.) 8–99 (Springer, 1993).

5. Cook, J. M. Sex determination in the Hymenoptera: a review of models and evidence. *Heredity* **71,** 421–435 (1993).

6. van Wilgenburg, E., Driessen, G. & Beukeboom, L. W. Single locus complementary sex determination in Hymenoptera: an ‘unintelligent’ design? *Frontiers in Zoology 2006 3:1* **3,** 1 (2006).

7. Heimpel, G. E. & de Boer, J. G. Sex determination in the hymenoptera. *Annu. Rev. Entomol.* **53,** 209–230 (2008).

8. Crespi, B. J. Heterozygosity in the haplodiploid Thysanoptera. *Evolution* **45,** 458–464 (1991).

9. Chapman, T. W., Crespi, B. J., Kranz, B. D. & Schwarz, M. P. High relatedness and inbreeding at the origin of eusociality in gall-inducing thrips. *Proceedings of the National Academy of Sciences* **97,** 1648–1650 (2000).

10. McLeish, M. J., Chapman, T. W. & Crespi, B. J. Inbreeding ancestors: the role of sibmating in the social evolution of gall thrips. *J. Hered.* **97,** 31–38 (2006).

11. Jordal, B. H., Normark, B. B. & FARRELL, B. D. Evolutionary radiation of an inbreeding haplodiploid beetle lineage (Curculionidae, Scolytinae). *Biol J Linn Soc* **71,** 483–499 (2000).

12. Hamilton, W. D. in *The Natural History of Inbreeding and Outcrossing* (ed. Thornhill, N. W.) 429–450 (University of Chicago Press, 1993).

13. Tang, X.-T., Tao, H.-H. & Du, Y.-Z. Microsatellite-based analysis of the genetic structure and diversity of Aleurocanthus spiniferus (Hemiptera: Aleyrodidae) from tea plants in China. *Gene* **560,** 107–113 (2015).

14. Hughes-Schrader, S. Cytology of hermaphroditism in Icerya purchasi (Coccidae). *Cell Tissue Res* **2,** 264–290 (1925).

15. Royer, M. in *Intersexuality in the animal kingdom* 135–145 (Springer, 1975).

16. Gardner, A. & Ross, L. The Evolution of Hermaphroditism by an Infectious Male-Derived Cell Lineage: An Inclusive-Fitness Analysis. *The American Naturalist* **178,** 191–201 (2011).

17. Adamson, M. Evolutionary patterns in life histories of Oxyurida. *Int. J. Parasitol.* **24,** 1167–1177 (1994).

18. Adamson, M. L. Evolutionary Biology of the Oxyurida (Namatoda): Biofacies of a Haplodiploid Taxon. *Advances in Parasitology* **28,** 175–228 (1989).

19. Crompton, D. W. T. & Nickol, B. B. *Biology of the Acanthocephala*. (Cambridge University Press, 1985).
